# Three-dimensional structural and metric characterisation of cardioids

**DOI:** 10.3389/fcell.2024.1426043

**Published:** 2024-07-25

**Authors:** Stefan H. Geyer, Lavinia Ceci Ginistrelli, Tobias Ilmer, Karoline M. Schwendt, Sasha Mendjan, Wolfgang J. Weninger

**Affiliations:** ^1^ Division of Anatomy, Center for Anatomy and Cell Biology, MIC, Medical University of Vienna, Vienna, Austria; ^2^ Institute of Molecular Biotechnology of the Austrian Academy of Sciences (IMBA), Vienna Biocenter, Vienna, Austria; ^3^ Division of Cell and Developmental Biology, Center for Anatomy and Cell Biology, Medical University of Vienna, Vienna, Austria

**Keywords:** heart, cardiac organoids, imaging, morphometry, cardioid

## Abstract

Exact three-dimensional (3D) structural information of developing organoids is key for optimising organoid generation and for studying experimental outcomes in organoid models. We set up a 3D imaging technique and studied complexly arranged native and experimentally challenged cardioids of two stages of remodelling. The imaging technique we employed is S-HREM (Scanning High Resolution Episcopic Microscopy), a variant of HREM, which captures multiple images of subsequently exposed surfaces of resin blocks and automatically combines them to large sized digital volume data of voxels sizes below 1 μm^3^. We provide precise volumetric information of the examined specimens and their single components and comparisons between stages in terms of volume and micro- and macroanatomic structure. We describe the 3D arrangement and lining of different types of cavities and their changes between day 10 and day 14 and map the various cell types to their precise spatial and structural environment. Exemplarily, we conducted semiautomatic counts of nuclei. In cryo-injured cardioids, we examined the extension and composition of the injured areas. Our results demonstrate the high quality and the great potential of digital volume data produced with S-HREM. It also provides sound metric and structural information, which assists production of native and experimentally challenged left ventricle cardioids and interpretation of their structural remodelling.

## 1 Introduction

Heart development is a highly complex morphogenetic process. In humans, it involves tight orchestration of cell migration, cell differentiation and remodelling of a linear heart tube into a four chambered organ. Even slight irregularities in the stringent spatio-temporal coordination of gene product interactions and alterations of biomechanical forces might cause severe defects (Buckingham et al., 2005; Bruneau, 2008; Kelly, 2023). Unsurprisingly, the heart is therefore the organ with the highest rate of congenital abnormalities, which often are causal to *intrauterine* death, reduced lifespan, and/or serious restrictions in life quality (Hoffman and Kaplan, 2002). As a consequence, there is an enormous socio-economic need for understanding the mechanisms of heart formation and for developing diagnostic, prevention, and therapeutic strategies.

Model organisms like the mouse, zebrafish, and chick offer the possibility to study the triggers and effects of morphogenetic events, and experimentally perturbed signalling pathways (Zaffran and Frasch, 2002; Kelly, 2023). However, studying heart formation in early embryos, especially of mammals, is challenging. Also, specific signalling pathways differ between models and humans, wherefore the transfer of results of gene knock-out and knock-in experiments from models to humans is problematic and doubtful (Bruneau, 2008; Kim et al., 2020; Hofbauer et al., 2021a). Finally, animal welfare strongly demands the search and use of alternatives to animal experiments.

An alternative to using animals for research emerged in the last decade, with the advent of methods for cultivating cells to form three-dimensional (3D) resemblances of early organs, organoids (Scalise et al., 2021). Such 3D cell cultures, composed of self-assembling human stem cells, therefore permit researching basic mechanisms of tissue and organ formation and remodelling, as well as response to external forces and tissue regeneration in a three-dimensional context. This permits investigation of human-specific processes involved in early organ development in order to explore fundamental mechanisms underlying malformations and diseases (Kretzschmar and Clevers, 2016; Bredenoord et al., 2017) and examination of tissue response after experimental damages in order to study tissue repair and regeneration (Linnemann et al., 2015; Grassi et al., 2019). To assist cardiovascular research, self-organising cardiac organoids have been recently established. These cardioids recapitulate early heart development by forming chamber-like structures comprised of myocardial and endothelial cells. In parallel to the development of methods for creating cardioids, methods have been adapted to challenge their integrity to research the fundaments of heart repair (Hofbauer et al., 2021a; Hofbauer et al., 2021b).

Analysing the morphology, tissue architecture, and composition of organoids is the key for evaluating and optimising the generation procedure and quality of organoids. Also, it is crucial for interpreting the effect and outcome of gene manipulation and injuries. Since organoids are 3D structures, conventional two-dimensional (2D) imaging methods, such as traditional microscopy do not provide satisfying information. 3D imaging methods are required. However, organoids are rather small, which precludes the use of the most popular 3D imaging techniques for assessing the morphology of organs in embryos, micro-magnetic resonance imaging (µMRI) and micro-computed tomography (µCT). These techniques, although providing 3D information, do not generate data in a resolution and tissue contrast, which allows precise visualisation of the rather small accumulations of heterogeneously differentiated cells composing organoids. Alternatives, such as confocal and light sheet microscopy have proven highly effective in producing 3D data. They provide cellular resolution, sufficient contrast, and allow for detection of specifically labelled cells. Yet, they are bound to utilize tissue clearing and have to rely on either autofluorescence or specific fluorescent signals for achieving tissue contrasts to examine morphology and tissue architecture (Renner et al., 2020; van Ineveld et al., 2020; Cunniff et al., 2021; Duclos et al., 2021; Gonzalez et al., 2021; Beghin et al., 2022; Brenna et al., 2022; Kang et al., 2022; Rodriguez-Gatica et al., 2022; Moos et al., 2024).

Strangely, one 3D imaging method, high-resolution episcopic microscopy (HREM) has not yet been tested on cardioids, although it is optimised for visualising the morphology and tissue architecture of organic objects with typical volumes of <1–175 mm^3^ in numeric resolutions of 2 × 2 × 2 to 6 × 6 × 6 µm^3^ (Weninger et al., 2006; Mohun and Weninger, 2012b; Geyer et al., 2015; Geyer and Weninger, 2019). We therefore decided to adjust the original HREM method to allow label-free, holistic visualisation of cardioid structure. For this, we increased the resolution and quality of the digital data produced with block face imaging by using block scanning procedures for data generation and stitching algorithms for combining the single images. This study aims to introduce this S-HREM (Scanning High Resolution Episcopic Microscopy) method and to explore its capacity for imaging the morphology and tissue architecture of native left ventricle cardioids of various stages of remodelling and left ventricle cardioids, which had been subjected to cryo-injury for studying tissue regeneration. Further, it aims to explore which morphologic and metric information can be extracted from such data and to provide first results of anatomic in-depth analysis of cardioids generated for studying tissue regeneration.

## 2 Materials and methods

### 2.1 Generation of cardioids

Eight self-organising left ventricle cardioids were generated from WTC hiPS cell line as previously described (Hofbauer et al., 2021b) and kept in CDM medium containing Insulin (10 μg/m, Sigma/Roche, #11376497001). Half of the cardioids were cryo-injured at day 7 of differentiation by contact with liquid N_2_-cooled steel for 2 s, until approximately one-third of the cardioid was injured. All cardioids were then allowed to continue maturation and harvested at day 10 and day 14 of differentiation, 3 and 7 days after cryo-injury respectively, by fixing in 4% PFA/PBS for 1 h.

### 2.2 Specimen processing

**FIGURE 1:**
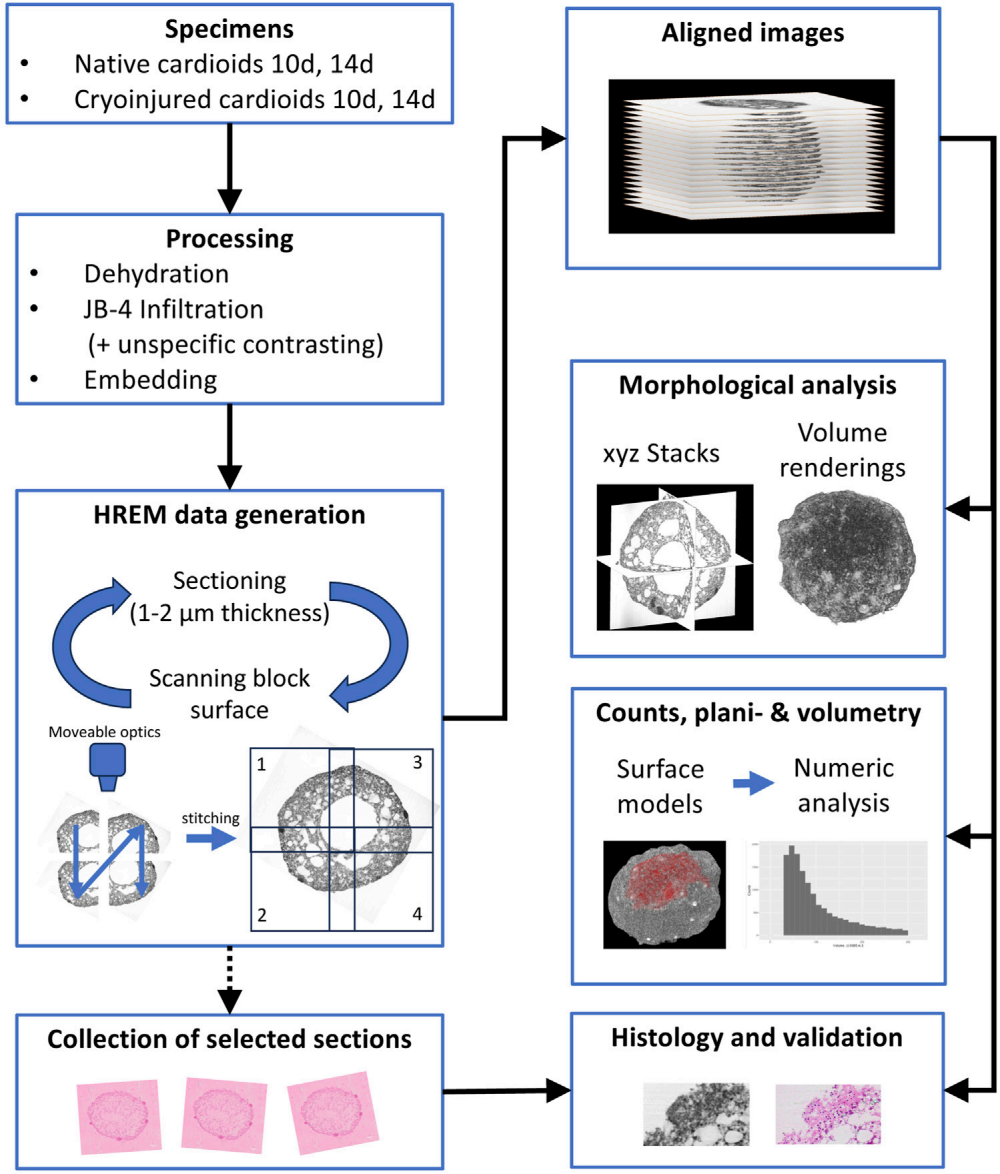
SHREM workflow.

All harvested cardioids were prepared for HREM imaging (Mohun and Weninger, 2012a; Geyer et al., 2017) according to a modified protocol, which addressed the small size and peculiar penetration conditions of cardioids (Figure 1). Fixed specimens were washed in phosphate buffered saline (PBS) for at least 12 h and dehydrated in a series of increasing ethanols (30%, 50%, 70%, 80%, 90%, 100%; 1 hour each) under constant rocking at 4°C. Then, the samples were infiltrated and embedded in dyed methacrylic resin JB-4 (Polysciences Inc., Warrington, PA, United States), prepared according to standard HREM protocols (Mohun and Weninger, 2012a; Geyer et al., 2017). This included immersion in JB-4 solution A of the JB-4 embedding kit, which contained 1.25 gm benzoyl peroxide catalyst and 0.4 g eosine B per 100 mL for 4 h under constant rocking at 4°C for infiltration. Then they were transferred into embedding moulds, filled with fresh embedding solution, consisting of 25 mL infiltration solution and 1 mL of solution B of the JB-4 embedding kit. After placing the block holders in the moulds, they were covered with mineral oil (Reichert Jung Schlittenbahnöl Nr. 404) to allow for oxygen-free polymerisation overnight at 4°C. After this, the blocks were put out of the moulds and stored for at least 2 days at room temperature to complete hardening of the resin.

### 2.3 HREM data generation

**FIGURE 2:**
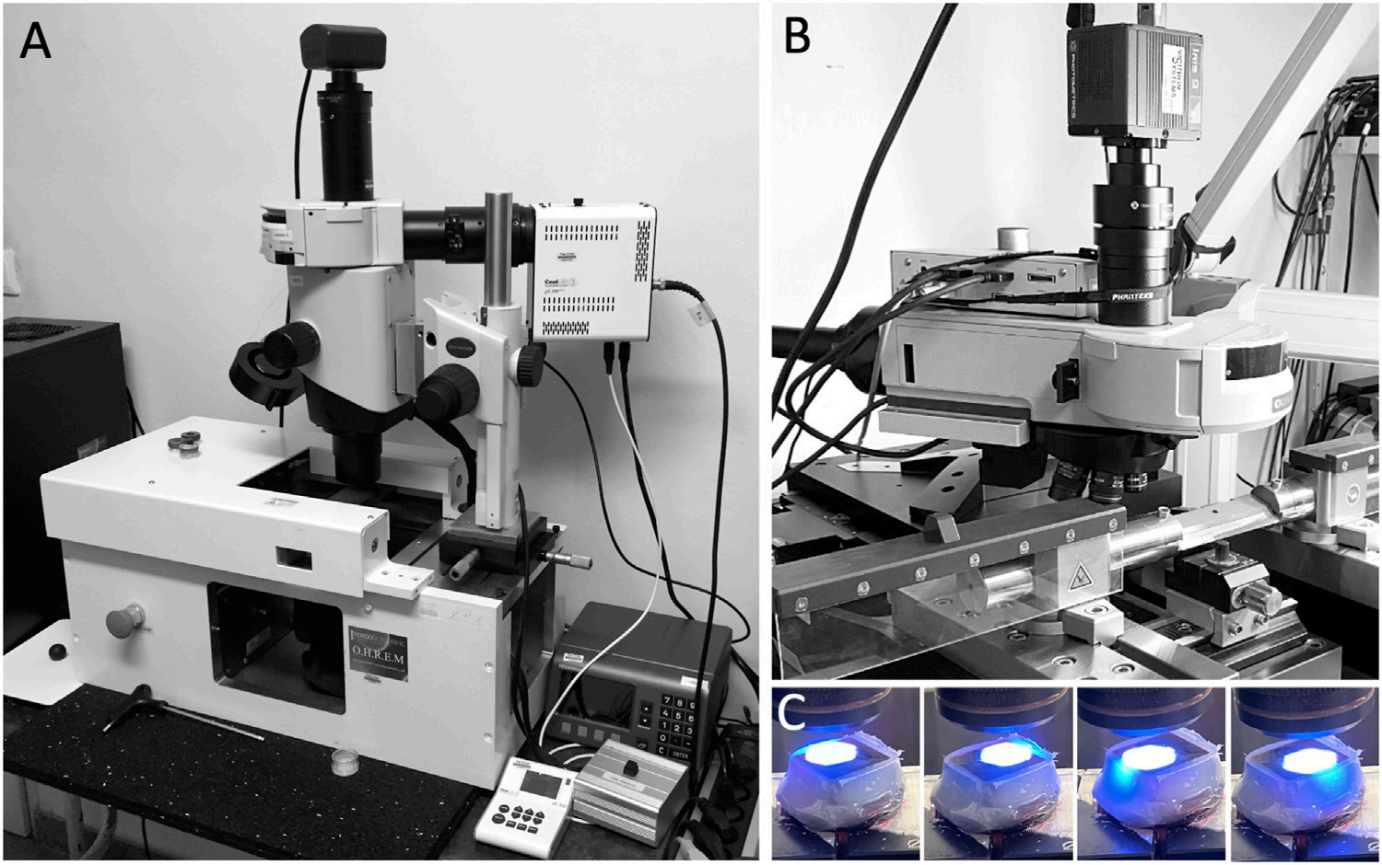
HREM **(A)** and S-HREM apparatus **(B)**. **(C)** Block face scanning.

Fully hardened JB-4 Blocks were mounted on a novel HREM prototype (Figure 2) and digital volume data were generated following the general principles of HREM data generation (Weninger et al., 2006; Mohun and Weninger, 2012c; Geyer et al., 2017).

The main hardware components of the HREM-apparatus were a sliding microtome (Microtome-L, microTec Laborgeräte GmbH, Walldorf, Germany); an Olympus BXFM microscope, equipped with a changeable filter cube containing a GFP filter (excitation 470/40, emission 575/50) and shutter; a x-y cross table (GT8-NSMA, Walter Uhl technische Mikroskopie GmbH & Co. KG); an external LED light source (pe-4000 High Power LED system); an IRIS-9 CMOS camera, and a PC equipped with 32 GB RAM and high end graphic card.

The components were arranged as follows: The optics with camera were positioned on a motorised cross table with the optical path directed vertically to target the center of the photoposition of the microtome. Optics, cross table, microtome, and camera were connected to the PC and the activity of the single components was synchronised by a customised software package on the basis of the VisiView 6 software (Visitron Systems GmbH, Puchheim, Germany).

This set up permitted creation of HREM data in a fully automated way following a strict protocol (Figure 1). In brief, the surface of the JB-4 block mounted on the microtome was scanned by capturing four neighbouring grey scale images of each freshly cut block surface, by using a 10x objective with a numeric aperture (NA) of 0.4. The neighbouring block face images were then digitally stitched to form a single image with the left upper corner as reference point and stored on a hard drive. Image size was 5,624 × 5,624 Pixels; numeric resolution was 0.42 × 0.42 µm^2^. Then a 2 µm thick (1 µm in one specimen) section was cut and block face scanning of the freshly exposed block face was performed again. The process of sectioning and block face scanning was repeated until the embedded specimen was fully sectioned. Such, series of 300–600 high resolution aligned block face images were created. The majority of the physical sections was discarded, but selected physical sections were preserved at regular intervals from four specimens.

### 2.4 HREM data visualization and 3D analysis

Loading the image series into the software Amira Version 2022 (Thermo Fisher Scientific, Merignac, France), the images were virtually stacked to digital volume data with voxel dimensions of 0.42 × 0.42 × 1 μm^3^ and 0.42 × 0.42 × 2 μm^3^ respectively. They were used for 2D and 3D analysis employing virtual dissection, surface and volume rendering algorithms and metric tools of the Amira software package. Total time from starting HREM data generation to starting 3D analysis of volume rendered specimens was between 2 and 4 h.

The surface and volume of all cardioids, the cumulative volume of their tissue components and empty cavities, and the diameters of their central cavities were measured. Furthermore, the numbers of the densely packed accumulations of cells were counted and the volume of each accumulation was determined. In the cryo-injured specimens the surface, thickness, and volume of the damaged area were measured and in one day 14 cardioid, the number and single volumes of the isolated spherical cavities were determined. Finally, the number of cell nuclei were counted in all day 10 cardioids and in one of them the diameters of the nuclei were measured.

Numeric and metric analysis of surfaces and volumes were performed on surface rendered objects, which were generated by using automated or semi-automated contour finding tools. The diameters of the central cavities were measured by selecting the image with the largest visible diameter from the stack of S-HREM images and measuring the distances by employing the two-dimensional (2D) measuring tools of the Amira software. The diameters of cell nuclei were measured by following a similar approach, but only the largest diameter of each nucleus within a representative mantel layer volume of 10^6^ μm^3^ was measured.

### 2.5 Traditional histology

The physical resin sections collected during S-HREM data generation were placed in a water bath (37°C) and mounted on glass slides. The mounted sections were washed with distilled water for 30 s, stained with Mayer’s haematoxylin for 20 min, rinsed in running tap water for 5 min, stained with eosin B for 25 min, and rinsed again in running tap water for 2 min. After washing in distilled water for 2 × 1 minutes, they were dried at room temperature and cover slipped with a standard mounting medium (Entellan, Merck Millipore, Temecula, United States). From these sections, digital images were captured with an Olympus V120-S5 slide scanner using a 20x lens (NA 0.75). Comparisons between S-HREM images and corresponding physical sections permitted quality assessment and served for evaluating data accuracy, quality and interpretation.

## 3 Results

**FIGURE 3:**
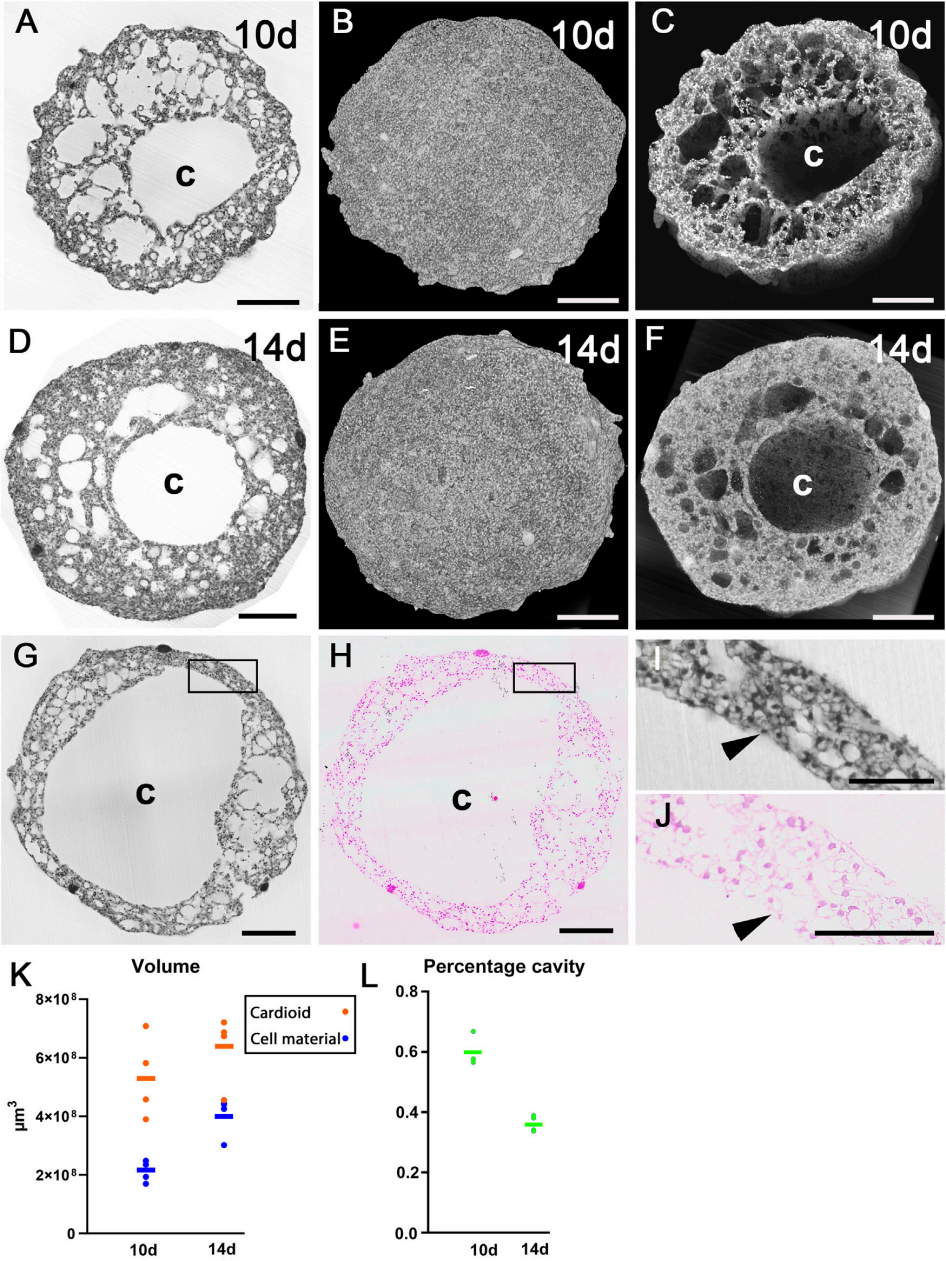
Architecture of cardioids. **(A–C)** HREM data of 10d cardioid. Single HREM-section **(A)**. Stacks of sections displayed as volume rendering **(B)** and volume rendering clipped **(C)** at position of section shown in **(A)**. Note the dimension of the central cavity **(C)**. **(D–F)** HREM data of 14d cardioid. Single HREM-section **(D)**. Stacks of sections displayed as volume rendering **(E)** and volume rendering clipped **(F)** at position of section shown in **(D)**. Note the dimension of the central cavity **(C)**. **(G–J)** Validation of HREM section quality. 10d cardioid. Note the resemblance of the HREM section **(G)** and the corresponding H&E-stained histological section in overview **(G,H)** and detail **(I,J)** and the lining of the central cavity (arrowheads, **(I,J)**. **(K)** Total cardioid volume (red) and volume of cell material excluding cavities (blue) in 10d and 14d cardioids. **(L)** Percentage of cavities on total volume. Scale bars 250 µm.

All eight cardioids were almost spherical (Figures 3A–F). The total volume of those harvested at day 10 was on average 0.535 mm^3^ (range 0.390–0.709 mm^3^). The volume of those harvested at day 14 was 0.634 mm^3^ (range 0.455–0.720 mm^3^). The total volume of material was 0.212 mm^3^ (range 0.170–0.248 mm^3^) in day 10 cardioids and 0.403 mm^3^ (range 0.302–0.444 mm^3^) in day 14 cardioids (Figure 3K). The rest was occupied by cavities. The core of the cardioids were voluminous, mostly spheroid-shaped cavities (Figures 3A,C,D,F,G,H) with an average diameter of 574 µm (range 449–812 µm) in cardioids of day 10 and 530 µm (range 308–682 µm) in cardioids of day 14. This was 59% of the total diameter in the day 10% and 51% of the total diameter in the day 14 cardioids. The cavity was largely lined by a single layer of flat to cuboid cells, although, at some locations, such a layer was missing (Figures 3G–J).

### 3.1 Mantle layer

**FIGURE 4:**
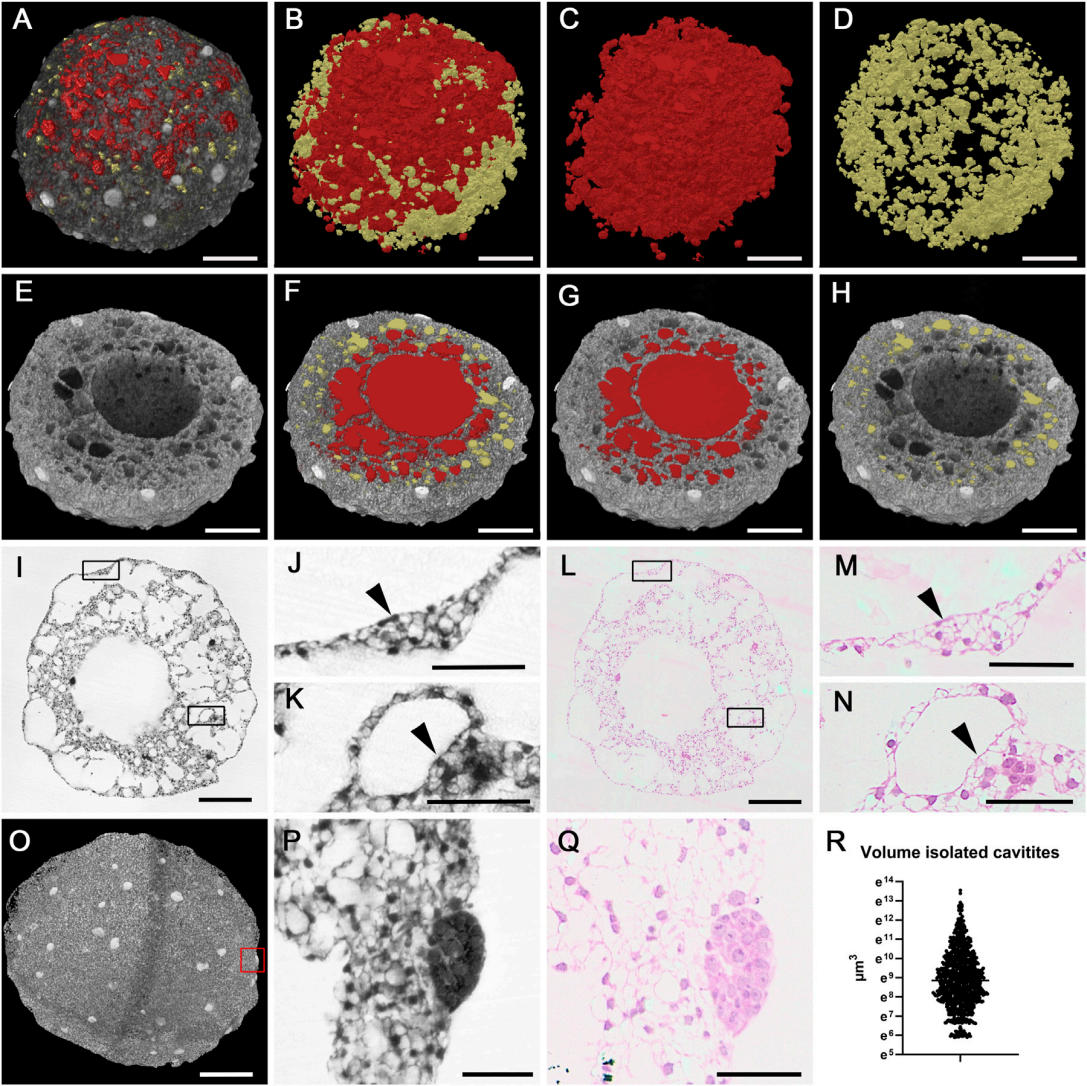
Cavities of a 14d cardioid. **(A–D)** Cavities of mantel layer. Cavities connected with central cavity displayed as red surface models and isolated cavities displayed as yellow surface models in A in combination with semitransparent volume rendering of the cardioid. **(E,F)** Central cavity (red) and mantel layer cavities in virtually clipped HREM data displayed in volume rendering **(E)** and combined volume and surface rendering mode **(F–H)**. **(I–N)** Cellular lining of cardioid surface [details **(J,M)**; arrowheads] and spherical holes **(K,N)**; arrowheads in original HREM-section **(I–K)** and corresponding H&E-stained histological section **(L–N)**. Boxed area in I and L are magnified in **(J,K,M,N)** respectively. **(O–Q)** Globe shaped cell accumulations. Volume rendering of a 10d cardioid. HREM section **(P)** and corresponding H&E-stained section **(Q)** of globe shaped accumulation boxed in **(O)**. Corresponding H&E-stained section **(Q)**. **(R)** Scatter plot of volumes of isolated cavities in a 14d cardioid. Scale bars 250 µm in **(A–I,L,O)**, Scale bars 50 µm in **(J,K,M,N,P,Q)**.

The surrounding of the central cavity was named as “mantle layer”. It was chiefly composed of irregularly shaped cells. With the exception of the damaged area in the four cryo-injured specimens, the superficial parts of this layer also comprised flat and cuboid cells (Figures 4I,J,L,M). This was confirmed by examining physical S-HREM sections with a traditional light microscope at a magnification of ×20 (Figure 4M). In the 4 day 10 cardioids the total number of cell nuclei ranged between 72,000 and 80,000 (mean 77,000). The mean diameter of the nuclei in a randomly selected mantel layer volume of 10^6^ μm^3^ was 6.49 µm (standard deviation = 0.635 µm).

The material of the mantle layer was interspersed with numerous holes of various diameters. Some of these holes were spheroid. Others were irregularly shaped (Figures 4A–H).

In depth analysis by eroding 3D-models with virtual clipping algorithms revealed connections between the central cavity and almost all of the irregularly shaped holes as well as interconnections between neighbouring ones (Figures 4E–H). On visual inspection, day 10 cardioids appeared to have looser tissue and much more cavities than day 14 cardioids. This observation was also reflected by volumetric analysis, which showed that the total volume of the central cavity and the tissue free spaces scattered in the mantle layer on average made up 60% of the volume of day 10 cardioids, while they only made up for approximately 36% of the volume of day 14 cardioids (Figure 3L).

The spherical holes of the mantle layer were fully isolated. They neither had connections to other holes, nor to the central cavity. These holes were evenly distributed and, by visual exploration of the original S-HREM section images, always lined by a single layer of flat to cuboid cells (Figures 4K, N). Exemplary binarisation of these holes in a day 14 cardioid followed by automatic counts and quantification showed that their total volume accounted for 0.021 mm^3^. This equalled 3% of the total volume of this cardioid. The number of these holes was 909 and their mean volume was 23,591 μm^3^ (363 μm^3^ to 763,270 μm^3^) (Figure 4R), which equals a diameter of 35 µm.

Describing the situation of the mantle layer from the point of the cellular material rather than from the point of the empty spaces: The biological material formed trabecular like structures, separated by irregular extensions of the central cavity. Inside the trabecular like structures, spherical holes were located. In day 10 cardioids the entire mantle layer was composed like this. In day 14 cardioids only the inner parts of the mantle layer had this appearance, while the superficial part of the mantle layer was a relatively compact layer of cells, in which some isolated and spheroid holes were dispersed (Figures 3C, F).

Prominent features, predominantly located near the outer surface of the mantle layer, were roughly globe shaped accumulations of small cells with dense cytoplasm. These accumulations had a mean volume of 23,727 μm^3^ (2,555 μm^3^–126,471 μm^3^) in day 10 cardioids and 90,712 μm^3^ (1726 μm^3^–436,734 μm^3^) in day 14 cardioids (Figures 4O–Q).

### 3.2 Site of cryo-injury

**FIGURE 5:**
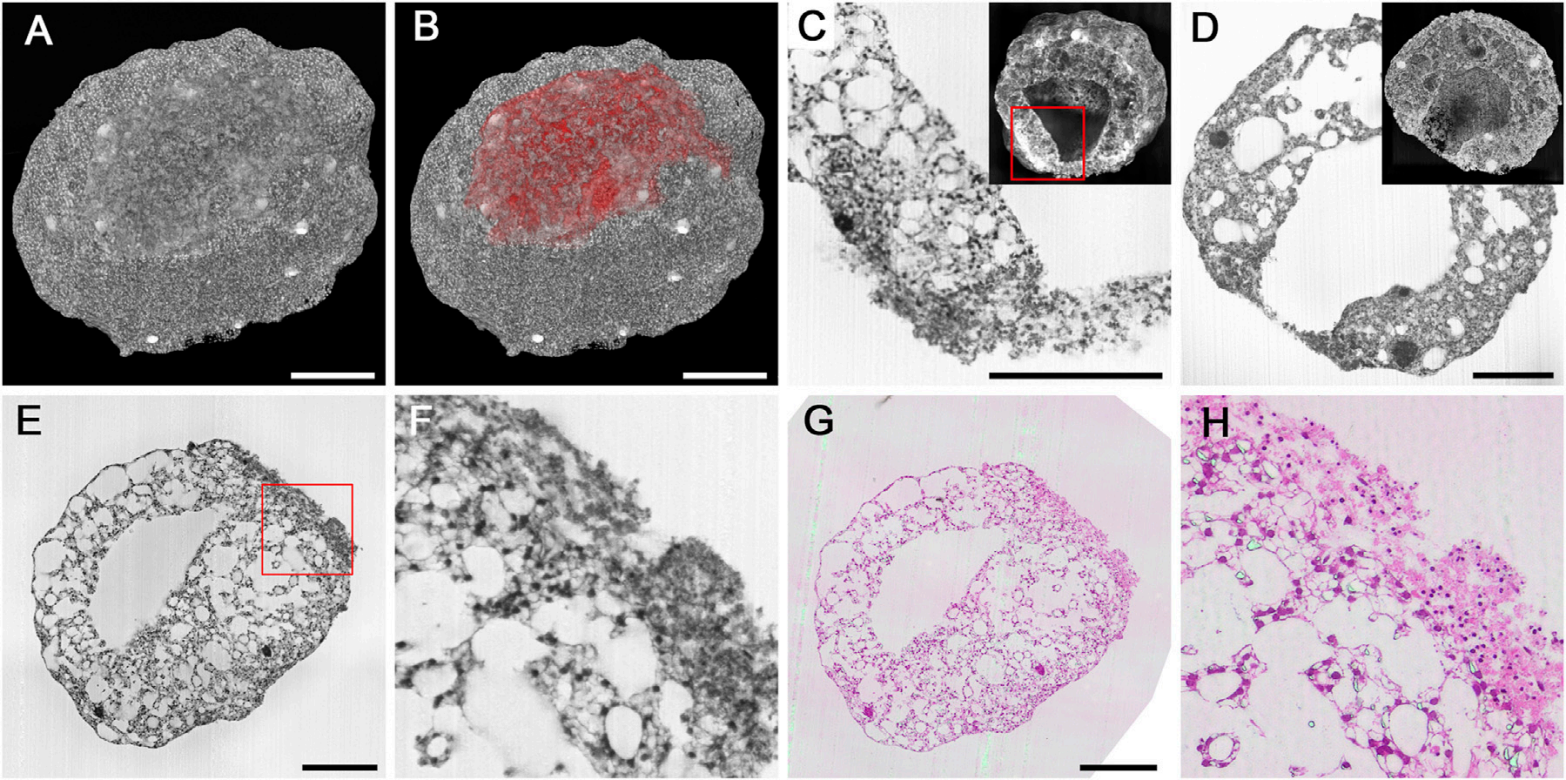
Cryo-injured cardioids. **(A,B)** Surface defect in a 10d cardioid, displayed in B as red surface model combined with a semitransparent volume rendering. **(C,D)** Details of injured mantel layer in the transition zone to normal tissue **(C)** and in the center of the injured region in a 14d cardioid. HREM sections and virtually cut volume models as inlays. Note the pyknotic nuclei in the injured region and the underlying normal mantel tissue adjacent to injured tissue in C. **(E–H)** Validation of HREM quality in displaying features of the injured region. Original HREM section **(E,F)** and corresponding H&E-stained histological section **(G,H)** in overview **(E,G)** and detail **(F,H)**. Scale bars 250 µm.

Cryo-injury had caused tissue damage in an almost circular area of 0.57 mm^2^ (17% of the total surface) in day 10 and 0.84 mm^2^ (21% of the total surface) in day 14 cardioids (Table 1). The central region lacked all cells regularly being present in the mantle layer (Figures 5A, B). Instead, merely cloddy material composed of tiny cells with pyknotic nuclei and indifferent extracellular material was visible (Figures 5C–H). The average thickness of the layer of cloddy material was 100 μm at day 10 and 108 μm at day 14. The volume of the cloddy material was 13% of the total tissue volume in day 10% and 12% of the total tissue volume in day 14 cardioids.

**TABLE 1:** Total surface area and damaged surface area of cryo-injuried cardioids (n = 4).

(d)		Surface area of cardioid (µm^2^)	Area of injury (µm2)	% Injury area
10	Cardioid 1	3,495,522	680,903	19.48
	Cardioid 2	3,239,176	451,629	13.94
	mean	3,367,349	566,266	16.82
14	Cardioid 3	3,452,962	925,259	26.80
	Cardioid 4	4,698,834	749,511	15.95
	mean	4,075,898	837,385	20.54

In the periphery of the injured area, normal mantle tissue was located beneath the damaged tissues. In the transitional zone of injured and normal mantle layer, cells with intact cell nuclei were dispersed in dense extracellular material. Parts of the injured areas were discontinuous or ruptured, which allowed direct connection of the central cavity of the cardioid with the outside (Figure 5D).

## 4 Discussion

Organoids are a novel and excellent alternative to the use of animal models for researching basic genetic concepts of tissue formation, remodelling, and repair. They are essentially comprised of pluripotent human stem cells or adult stem cells, which are chemically and physically forced to differentiate and form 3D cell accumulations of increasing complexity (Lancaster and Knoblich, 2014; Artegiani and Clevers, 2018; Kim et al., 2020; Scalise et al., 2021). The composition and architecture of organoids is heavily influenced by the creation process. Even minimal alterations in the composition of the cultivation media, the sequence of the addition of growth factors, or the structure of the extracellular matrix, may cause serious effects resulting in an enormous morphological heterogeneity (Little and Combes, 2019; Scalise et al., 2021; Beghin et al., 2022). Therefore, constant evaluation of the structural architecture and tissue composition of organoids during their formation and examining the effect of changing various conditions are essential companions of organoid production.

Imaging modalities that were already adopted for analysing the architecture and composition of organoids are conventional high-end two-dimensional microscopy (Norrie et al., 2021; Charlet-Faure et al., 2023) as well as a larger spectrum of 3D imaging methods, such as light sheet microscopy, confocal microscopy, and optical coherence tomography (van Ineveld et al., 2020; Beghin et al., 2022; Keshara et al., 2022; Morishita et al., 2023). Especially light sheet microscopy proved to be of high accuracy and scientific value–and the data even permit quantifications of single cells using (auto-)fluorescence signals (Dekkers et al., 2019; Albanese et al., 2020).

As a matter of fact, all imaging techniques have to trade-off between high data resolution and a large field of view, respectively volume (Weninger and Geyer, 2008; Park et al., 2021). This often precludes simultaneous exploration of cell details, 3D architecture, and overall morphology and hinders metric analysis. Hence, especially the comprehensive visualisation and examination of highly complex organoids, such as cardioids, which are derived from induced human pluripotent stem cells (hiPSC) and are considered to develop ventricular cardiac tissue (Hofbauer et al., 2021a; Hofbauer et al., 2021b) is challenging. Since we received excellent results when imaging small to late chick and mouse embryos with the HREM imaging method (Liu et al., 2014; Weninger et al., 2018; Geyer et al., 2022; Reissig et al., 2022), we decided to modify HREM by implementing block scanning into the data generation process. This allows to capture information from a larger field of view, while being able to use high resolution objectives with optimal numeric apertures for image capturing. We named this modified HREM version S-HREM and decided to use it for examining eight cardioids; four harvested on day 10 and four harvested on day 14; half of them challenged by cryo-injury. As our results demonstrate, the S-HREM data excellently fitted for structural and metric analyses of morphologic features and for spatial mapping of the cell types that can be distinguished according to traditional histologic criteria. Due to the good results, we are confident that S-HREM will likewise permit highly detailed examinations of all types of organoids, as well as all sorts of structurally complex specimens of similar volumes.

All examined cardioids showed a voluminous, mostly spherical empty cavity in their centres. This was surrounded by a mantle layer composed of cell accumulations and interspersed with two types of small empty cavities of different dimension and shape. One type was connected to the central cavity and exhibited an irregular inner surface. The second type was fully isolated, spherical and lined by flat to cuboid cells. While our exemplary measurements prove that the volume of the latter can be measured in a semiautomated way, the continuity with the central cavity prevented mathematic definitions of the dimensions and surface of the first type. Both types of cavities can be clearly distinguished from artifacts. The globe like isolated ones, because of the cells lining the inner surface, the irregular shaped ones because of their continuation with the central cavity.

Earlier studies have also described empty cavities as regular features of cardioids and postulated that they progressively coalesce to form larger cavities (Hofbauer et al., 2021b). Our results support this idea and add first volumetric and structural information on these cavities. However, large scale analysis of cardioids harvested in a tight time sequence and careful in-depth analysis are required to fully prove this concept.

Volumetry revealed that day 10 cardioids are only slightly smaller than day 14 cardioids, although the latter are composed of approximately twice as much material. As our data explain, this is mainly due to the reduction of the volume of the central cavity and increasing compactification of the superficial parts of the mantle layer. This is in line with the maturation protocol of cardioids, with matured cardioids having a smaller cavity and thicker cell layer (Schmidt et al., 2023). However, the increase of cellular material at the cost of the volume of the internal cavity is unexpected. We would have anticipated that increasing the cellular material of the mantle layer causes a substantial increase of the total volume between day 10 and 14 and that the volume of the central cavity, which is considered as the cavity of a forming ventricle, remains more or less constant. A hypothetical explanation worth be tested is that physical forces caused by dense connections of the cells, or their membranes, forming the continuous border covering the mantle layer, restricts expansion of the cardioid and forces the newly created cells to occupy spaces inside.

S-HREM data proved to be of sufficient resolution to visualise single cell shape morphology in the context of whole cardioids. This enabled us to map the spatial location of four morphologically distinguished types of cells. Firstly, irregularly shaped cells: These were the principle cellular component of the closely-packed and the trabecular like material in between the irregularly shaped holes of the mantle layer. Earlier studies identified them as cardiomyocytes (Hofbauer et al., 2021b). Their irregular shape is the result of shrinkages introduced during specimen processing (dehydration, infiltration) for S-HREM embedding. However, it has to be emphasised that shrinkages and other specimen processing artefacts are inherent to all histology-based methods. Secondly, flat cells: They were located near the surface of the cardioids with the exception of the site, where those cardioids that had been cryo-treated were injured. Here, their external cell membranes formed a continuous layer. In addition, flat cells, together with a small portion of cuboid cells lined large sections of the internal surface of the central cavity and its irregular extensions into the mantle layer. Thirdly, cuboid cells: They contributed to the lining of the isolated, spherical cavities distributed in the mantle layer. Together with the flat ones, these cuboid cells might be considered as forerunners of endothelial cells. This is in line with earlier findings, which describe endothelial cell differentiation and self-organisation controlled by WNT, ACTIVIN and VEGF (Hofbauer et al., 2021b). Fourthly, cells with optically dense cytoplasm: They formed small globes, predominantly near the surface and are considered as endoderm cells. Such cell accumulations cannot be seen in day 30 cardioids (Schmidt et al., 2023). We thus assume, they undergone differentiation between day 14 and day 30 and that they maybe are linked to the formation of the global like cavities. To make sure that the information we provide on cell shapes is correct, we confirmed the cell shape analysis by direct comparisons between digital S-HREM images and their precise physical counterparts.

S-HREM faces the same issues as HREM (Reissig et al., 2021), wherefore original S-HREM images show artefacts, inherent to images produced with all types of microscopic imaging techniques. In particular, S-HREM block face images show slight inhomogeneities in tissue contrasts due to uneven illumination during image capturing–e.g., usually, the center of an image is brighter than its periphery. Under such conditions, threshold algorithms binarise only the dark centers of nuclei in the center of the images, while they binarise the entire nucleus material - or even artificially expand it - in the periphery. The fact that S-HREM images are composed of several smaller images, which are stitched, aggravates this problem. Furthermore, the presence of scratches in HREM block face images, which change unpredictably from image to images make it impossible to solve this problem, by using software or “blank field” approaches (Reissig et al., 2021).

To overcome this obstacle and to allow correct detection of all nuclei, we split the original (stitched) S-HREM image stacks into several sub-stacks, which were homogenous enough to define and execute thresholds for binarizing all nuclei. After this, we merged the sub-stacks and performed surface rendering. This enabled us to count all nuclei of day 10 cardioids. For day 14 cardioids, even this approach was not successful, since the contrasts between nuclei and surrounding plasma were too low. We thus conclude that, although S-HREM data do fit for counting nuclei in early cardioids, they do not fit for performing quick nuclei and cell counts in later cardioids.

Artificial shrinkage or expansion of anatomic structures during their automated segmentation is not a specific of data generated with microscopic imaging techniques. It is an ubiquitous phenomenon and often prevents meaningful volumetry of structures with small volumes (Ali et al., 2011). Thus, although S-HREM data allowed automated nuclei counts in day 10 cardioids, fully automated volumetry of the nuclei was prevented. Hence, we decided not to provide information of the volumes, but to provide measurements of the diameters of the nuclei and to measure them by using a semi-traditional approach. We selected a representative volume of the mantel layer, scrolled through the stack of S-HREM images which comprises the data set, identified for each nucleus the very section in which it had its largest extension and measured the diameter with the aid of planimetric measuring tools. Although this approach is simple and generates significant information on nucleus size, it is time and labour consuming. Analyses of nucleus diameters on the basis of S-HREM data should therefore be reserved for special circumstances.

Various methods exist for challenging the integrity of cardioids to study regenerative responses in heart tissue (Voges et al., 2017; Hofbauer et al., 2021b). We chose to test the capacity of S-HREM on cardioids that were subjected to cryo-injury and to characterise the morphology of the damaged area. We observed, that all cryo-injured cardioids featured a clearly demarcated damaged area. Structural analysis of the border zone of undamaged and damaged tissue and accurate planimetric measurements of the extension of the defects proved to be possible and were conducted. Intriguingly the damaged area was slightly larger in day 14 cardioids than in the day 10 cardioids (21% versus 17%). This difference might be the result of either the slightly larger volume of the day 14 cardioids, or the use of different parameters while performing the injury. Since the aim of our study was restricted to providing first information on normal left ventricle and injured left ventricle cardioids and to exploring the capacity of S-HREM to characterise them, the injury process was not meticulously monitored. Hence, this study cannot provide an explanation for the observed difference. However, it impressively shows that S-HREM permits quick imaging and comprehensive characterisation of the impact of cryoinjury in terms of extension and tissues alterations. Furthermore, it shows that S-HREM data permit comprehensive examination of the peculiar morphology and tissue characteristics of the border-zone in between damaged and native material.

Technically, S-HREM is an expansion of the original HREM method (Weninger et al., 2006; Mohun and Weninger, 2012c; Geyer and Weninger, 2019). It permits 3D analysis of digital volume data of cardioids within 3–4 h after starting the data generation process. Similar to HREM the data generation set up comprises a microtome reaching a photo position after each cut, a fluorescence optic, which is aligned to this photo position and a digital camera, which sits on the optics and connects to a PC. These components are synchronized and operated by a customized image capturing software. The novelty of S-HREM is that the steering software not simply captures block face images. It also orchestrates scanning of the block surface by capturing multiple images that are finally stitched to form a single block face image. This is executed by shifting the optics with the aid of a cross table along defined distances across the desired field of view. The benefit of scanning the block face instead of simply capturing a single block face image is that high-end objectives with higher numeric apertures (in this study a 10 times objective with a NA of 0.4) can be used for image capturing. Such, a large field of view can be visualised in excellent contrast and high resolution; in our study a field of view of 1.5 × 1.5 mm^2^ was depicted on 12,755,102 Pixels. However, larger field of views in even higher resolution are possible with the S-HREM set up.

Due to the excellent data quality, S-HREM proved to be sufficient for characterising cell morphology and defining cell types by using traditional histologic criteria. This enabled precise spatial mapping of the single cells in respect to the cardioids and their tissues. - The correctness of the interpretation of cell morphology was confirmed by direct comparisons between images of S-HREM sections and images captured from haematoxylin/eosin-stained sections, collected after capturing the corresponding S-HREM block face image (Keuenhof et al., 2021). In addition, S-HREM data proved to allow comprehensive analysis of overall morphology and tissue architecture and to permit highly accurate quantitative analyses of anatomical structures. However, preparation of specimens for S-HREM imaging requires their fixation, dehydration and infiltration. Similar to specimen processing for traditional histology, this causes inhomogeneous shrinkages of tissues. This has to be considered when interpretating metric information, cell shapes, and tissue arrangement (Reissig et al., 2021). Also, S-HREM, as an *ex vivo* imaging method, does not allow 4D monitoring of developmental changes or regenerative processes. *In vivo* imaging methods such as optical coherence tomography (OCT) will allow this (Deloria et al., 2021), but at the cost of data quality. A possibility to profit from the advantages of both, *in vivo* and *ex vivo* imaging, is to combine OCT and HREM for examining identical specimens. The principle set up for an OCT-HREM imaging pipeline was already established a decade ago for chick embryos (Liu et al., 2014) and suggests, that such a multimodal approach can be also easily established for examining cardioids. OCT could be employed to monitor cardioid formation and to identify specimens of special interest, which are then harvested for detailed structural analysis with S-HREM. Designing and using this multimodal pipeline for analysing cardioids is bound to boost researching the basic principles of organ formation and tissue regeneration.

An important aspect of this study was to showcase the potential of S-HREM for analysing cardioids and to share the lessons learnt. In general, the preliminary results are in line with earlier studies (Hofbauer et al., 2021b), but S-HREM data revealed additional and important spatial information. This strongly endorses the design of in-depth studies, which systematically research homogenous batches of significant numbers of specimens to provide further insights into cardiac development and regeneration.

In a nutshell, the newly introduced S-HREM procedure enables highly detailed structural 3D examination and quick (volu)metric analysis of highly complex organoids, such as cardioids. First results of employing it for researching left ventricle cardioids and cryo-injured left ventricle cardioids at different stages of their differentiation, provide new information on cardioid architecture and lead to new hypotheses regarding cardioid maturation.

## Data Availability

The raw data supporting the conclusions of this article will be made available by the authors, without undue reservation.
